# Acromioclavicular Luxation with Fracture of the Lateral End of the Clavicle: Case Report

**DOI:** 10.1055/s-0043-1776015

**Published:** 2024-12-27

**Authors:** Evander Azevedo Grossi, Gustavo Heringer Cezar Fortes Silveira, Alessandra Bastos Borges

**Affiliations:** 1 Hospital Márcio Cunha, Fundação São Francisco Xavier, Ipatinga, MG, Brasil

**Keywords:** acromioclavicular joint, fracture fixation, joint dislocations

## Abstract

The present case report is an observational study with a literature review. This case is significant because the injury is rare regarding location and clinical manifestation. A middle-aged male patient sustained a fracture at the acromial end of the clavicle with lateral fragment dislocation after falling from a bicycle. The literature reports a single similar case, but we had no access to the complete text. The patient underwent treatment with satisfactory outcomes.

## Introduction

Acromioclavicular dislocation (ACD) occurs in 6% of dislocations. The second most common ACD is that of the shoulder girdle. 1 Acromioclavicular dislocation may present with clavicular fractures in its medial, middle, or lateral third. Acromioclavicular dislocation with clavicular lateral end fracture is an unusual injury, even more so when accompanied by a displacement of the lateral fragment of the clavicle to the acromion.

The main mechanism of traumatic ACD is falling onto the shoulder with the arm in adduction. This injury is 5 to 10 times more frequent in males. 2

Treatment varies according to the degree of dislocation and association with ipsilateral clavicle fractures. Surgical treatment options include fixation with a coracoclavicular screw, anchor ligature, Endobutton, and hook plate.

Rockwood 3 described six types of ACD. There are several classification methods for fractures of the lateral clavicular end, and the most cited is from Robinson. 3 Fracture of the lateral clavicular end associated with ACD is a rare condition, and it is not included in the proposed classification systems. It was only described by one author in English 4 and by another team in German. 5

Given the above, the present study aims to describe a rare case of fracture of the lateral clavicular end with dislocation of the fractured fragment to the trapezius musculature.

## Case Report

The patient, a 40-year-old man, sustained direct shoulder trauma after falling from a bicycle to the ground.

**Fig. 1:**
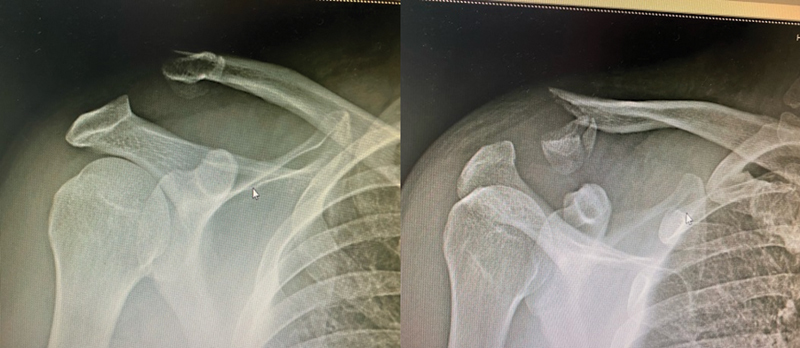
Radiograph showing the fracture of the lateral end of the clavicle with dislocation of this fragment away from the acromion and increased coracoclavicular space.

The initial clinical examination revealed intense local pain and a depressed shoulder. Radiographs showed a fracture of the lateral end of the clavicle, fragment dislocation and distancing to the acromion, and increased coracoclavicular space ( Fig. 1 ).

The patient underwent surgery after 2 days due to severe pain and local deformity. Through an incision over the acromioclavicular joint (ACJ) towards the clavicle diaphysis, we removed the bone fragment from the lateral end of the clavicle in the trapezius muscle. Next, we released the ligaments from the trapezius. We performed an osteosynthesis using Steinmann wires followed by ligation of these steel wires to a 5-mm anchor inserted in the coracoid with Fiber Wire 1 tied to the clavicle to reduce the coracoclavicular space. We did not perform ACJ fixation because of the fragmentation risk.

**Fig. 2:**
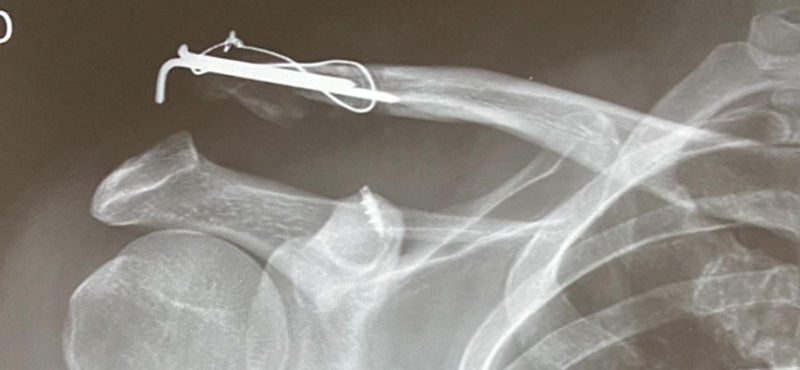
Radiograph revealing an increased coracoclavicular space due to rupture of the anchor wires and consequent new acromioclavicular dislocation.

Three weeks after surgery, the patient returned with shoulder pain and deformity, stating that it occurred after a physical effort. A radiograph showed an increased coracoclavicular space due to the rupture of the anchor wires and consequent new acromioclavicular dislocation ( Fig. 2 ).

**Fig. 3:**
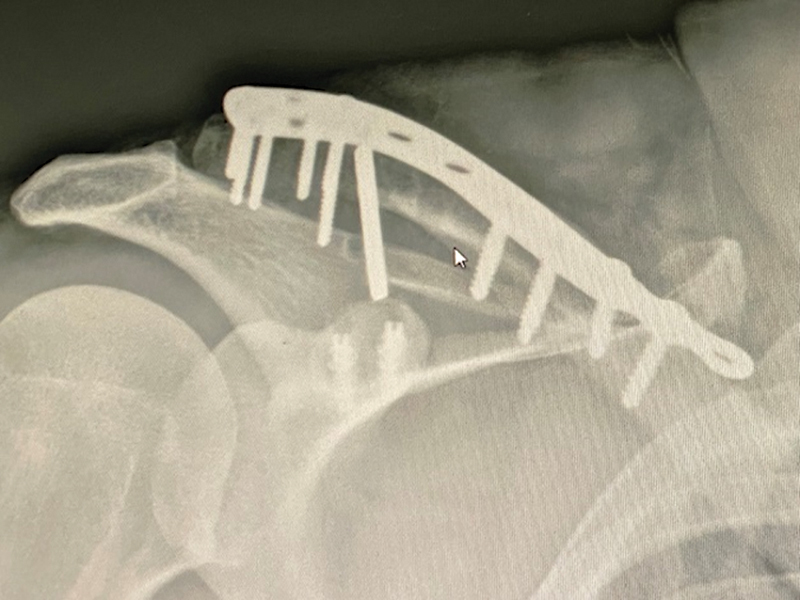
Osteosynthesis with a locked plate and new coracoclavicular ligature with another anchor tied over the locked plate. Fixation of the clavicle to the scapular spine with one Steinmann wire.

The patient underwent a second surgery to repeat the osteosynthesis. This time, we used a locked plate and performed a new coracoclavicular ligature with another anchor of the same type over the locked plate. To increase procedural safety, we fixated the scapular spine with one Steinmann wire, which we removed after 6 weeks ( Fig. 3 ).

**Fig. 4:**
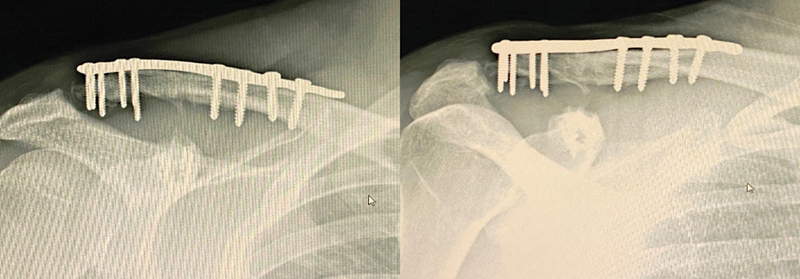
Radiograph 12 months after surgery showing fracture consolidation and no signs of osteonecrosis.

**Fig. 5:**
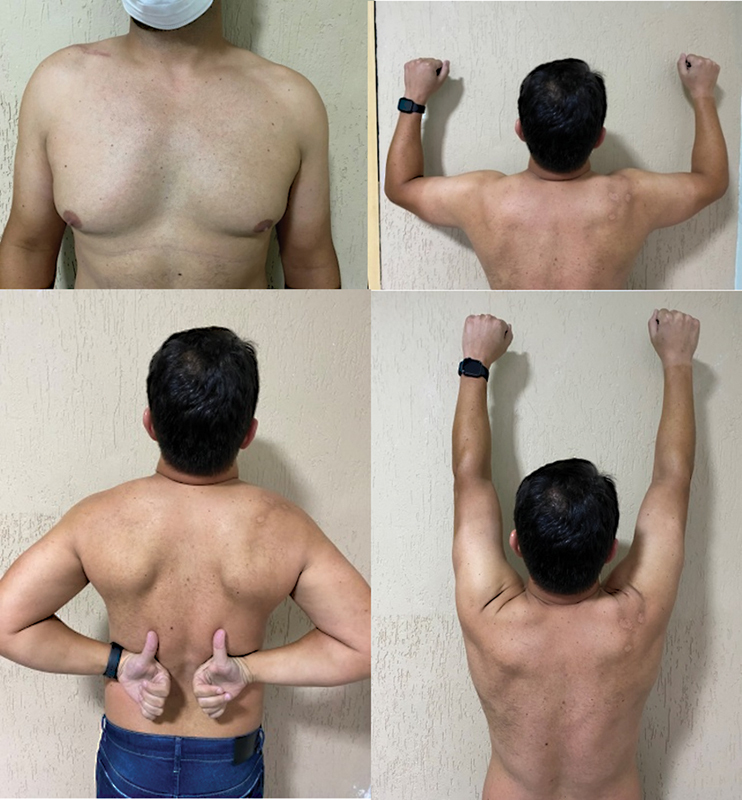
The clinical examination revealed a preserved, pain-free range of motion.

At the last follow-up, 12 months after surgery, the patient had no complaints of pain or functional loss. A new radiograph showed fracture consolidation and no signs of fragment osteonecrosis ( Fig. 4 ). Clinical examination revealed a preserved, pain-free range of motion ( Fig. 5 ).

## Discussion

We found only one article with the same case as that of our patient.

Borus et al. 4 describe a case identical to ours. However, we did not have access to their paper despite several attempts to obtain it, including requesting it via e-mail to the authors.

Siebenbürger et al. 5 published a case report in which the patient had a fracture of the lateral end of the clavicle and associated ACD. In their case, the fractured fragment did not completely deviate from the clavicle metaphysis, as in our patient. Thus, the treatment consisted of a closed fracture reduction and arthroscopic fixation with an Endobutton, resulting in a satisfactory outcome.

The literature describes ACDs with fractures of the diaphysis 6 7 8 and of the medial end of the clavicle 9 10 and several proposed treatments, but none is similar to our case.

The critical point is that, unfortunately, the patient required two surgeries due to the rupture of the anchor wires. We believe the loosening occurred due to the lack of accessory fixation. Therefore, during the second surgery, we changed the osteosynthesis method by using a locked plate and adding a ligation with an anchor. As such, we fixated the clavicle to the scapular spine, improving stability.

We believe fractures with displacement of the lateral end of the clavicle and associated injury to the acromioclavicular ligaments must undergo accessory fixation of the clavicle to the acromion or the scapular spine and not just ligatures to the coracoid process.
