# Supplementary material for: Acromioclavicular Luxation with Fracture of the Lateral End of the Clavicle: Case Report
Source: Rev Bras Ortop (Sao Paulo). 2024 Dec 27;59(Suppl 2):e203–6. doi: 10.1055/s-0043-1776015 (PMC11679612; doi:10.1055/s-0043-1776015)
Supplement: Related texts

# Research article: Luxação acromioclavicular associada a fratura da extremidade lateral da clavícula: Relato de caso

**Keywords:** articulação acromioclavicular, fixação de fratura, luxações articulares

## Abstract

O presente trabalho é uma pesquisa com delineamento observacional do tipo relato de caso, com revisão de literatura. A apresentação deste caso justifica-se por tratar-se de uma patologia com apresentação rara quanto à localização e manifestação clínica. É descrito um caso raro de um paciente masculino de meia idade que sofreu fratura da extremidade acromial da clavícula associada a luxação do fragmento lateral após queda de bicicleta. A literatura cita apenas um caso semelhante, mas não foi possível o acesso ao artigo completo. O caso foi discutido, tratado, e obteve resultado satisfatório.

## Introdução

A luxação acromioclavicular (LAC) ocorre em 6% das luxações, sendo a segunda mais comum na cintura escapular. 1 A LAC pode estar associada a fraturas da clavícula; porém, estas podem ser em seu terço medial, médio ou lateral. A ocorrência de LAC associada a fraturas da extremidade lateral é baixa; no entanto, quando há também um deslocamento do fragmento lateral da clavícula em relação ao acrômio, torna-se ainda mais infrequente.

O mecanismo do trauma da LAC é geralmente ocasionado pela queda sobre o ombro com o braço em adução. Sua frequência é de 5 a 10 vezes maior no sexo masculino. 2

O tratamento varia conforme o grau da luxação, e também de acordo com a associação de fraturas ipsilaterais da clavícula. Entre as opções de tratamento cirúrgico, são descritas fixação com parafuso coracoclavicular, amarrilho com âncora, Endobutton ou placa gancho.

A LAC é classificada em seis tipos de acordo com Rockwood. 3 Já as fraturas da extremidade lateral da clavícula têm várias classificações, entre as quais a mais citada é a de Robinson. 3 Fratura da extremidade lateral da clavícula associada a LAC é uma patologia muito rara e não está incluída nos sistemas de classificação propostos, sendo descrita apenas por um autor na língua inglesa 4 e outro em alemão. 5

Diante do histórico apresentado, o objetivo do presente trabalho é descrever um caso raro de fratura da extremidade lateral da clavícula com luxação do fragmento fraturado para a musculatura do trapézio.

## Relato do Caso

Trata-se de um paciente de 40 anos, sexo masculino, que sofreu queda de bicicleta com trauma direto do ombro ao solo.

O exame clínico inicial mostra muita dor local, deformidade com infradesnivelamento do ombro e exame radiográfico evidenciando a fratura da extremidade lateral da clavícula, luxação com distanciamento do fragmento em relação ao acrômio e aumento do espaço coracoclavicular ( Fig. 1 ).

**Fig. 1:** Exame radiográfico mostrando a fratura da extremidade lateral da clavícula com luxação deste fragmento para longe do acrômio, e aumento do espaço coracoclavicular.

Devido à dor intensa e deformidade local, o paciente foi operado após 2 dias. Foi realizado um acesso sobre a articulação acromioclavicular (AAC) em direção à diáfise da clavícula, retirado o fragmento ósseo da extremidade lateral da clavícula na musculatura do trapézio, que estava totalmente solto dos ligamentos, e foi feita a osteossíntese utilizando fios de Steinmann, amarrilho com fios de aço e inserida uma âncora de 5 mm no coracoide com fios Fiber Wire 1 amarrados à clavícula para redução do espaço coracoclavicular. Não foi realizada fixação da AAC devido ao risco de fragmentação.

Após 3 semanas de operado, o paciente retornou com dor e deformidade no ombro, informando ter ocorrido após um esforço físico. A radiografia apresentou aumento do espaço coracoclavicular por rompimento dos fios da âncora e consequente nova LAC, conforme radiografia ( Fig. 2 ).

**Fig. 2:** Radiografia apresentando aumento do espaço coracoclavicular por rompimento dos fios da âncora e consequente nova LAC.

Em seguida, o paciente foi reoperado, e então foi refeita a osteossíntese, dessa vez com placa bloqueada, novo amarrilho coracoclavicular com outra âncora do mesmo tipo associada a amarrilho feito sobre a placa bloqueada. Para maior segurança no procedimento, foi fixada a clavícula à espinha da escápula com um fio de Steinmann, o qual foi retirado após 6 semanas ( Fig. 3 ).

**Fig. 3:** Osteossíntese com placa bloqueada, novo amarrilho coracoclavicular com outra âncora, amarrada sobre a placa bloqueada. Fixação da clavícula à espinha da escápula com um fio de Steinmann.

No último retorno, 12 meses após a cirurgia, já sem queixas de dor e sem perda funcional, foi realizado novo controle radiológico que mostrou consolidação da fratura e sem sinais de osteonecrose do fragmento ( Fig. 4 ). Exame clínico mostrou amplitude de movimentos preservados e sem dor ( Fig. 5 ).

**Fig. 4:** Radiografia 12 meses após a cirurgia mostrando consolidação da fratura e sem sinais de osteonecrose.

**Fig. 5:** Exame clínico mostra amplitude de movimentos preservados e sem dor.

## Discussão

Nossa pesquisa encontrou apenas um artigo com o mesmo caso do nosso paciente.

Borus et al. 4 descrevem caso idêntico a este; porém, não foi possível acessar o trabalho, apesar de várias tentativas de obtê-lo, incluindo solicitação via e-mail para o autor.

Siebenbürger et al. 5 publicaram um relato de caso em que o paciente teve uma fratura da extremidade lateral da clavícula e luxação acromioclavicular associada, mas o fragmento fraturado não desviou totalmente da metáfise da clavícula, conforme o presente caso relatado. Assim, o tratamento realizado foi redução incruenta da fratura e fixação via artroscópica com Endobutton, obtendo resultado satisfatório.

A literatura descreve casos de LAC associada a fraturas da diáfise 6 7 8 e extremidade medial da clavícula, 9 10 com vários tipos de tratamentos propostos, mas nenhum deles se assemelham ao presente caso.

O ponto crítico é que o paciente infelizmente necessitou de duas cirurgias devido à intercorrência de rotura dos fios da âncora. Acreditamos que a soltura ocorreu por não haver nenhuma fixação acessória. Por isso, em sua segunda cirurgia, foi alterado o método de osteossíntese por placa bloqueada e além do amarrilho com âncora foi fixada a clavícula à espinha da escápula, obtendo assim melhor estabilidade.

Acreditamos que as fraturas com desvio da extremidade lateral da clavícula e lesão associada dos ligamentos acromioclaviculares devem ter fixação acessória da clavícula ao acrômio ou à espinha da escápula, e não somente por meio de amarrilhos ao coracoide.
